# Sodium caseinate hinders chymosin-induced aggregation of caseins in concentrated milk: The role of soluble caseins and calcium ions

**DOI:** 10.3168/jdsc.2021-0124

**Published:** 2021-09-23

**Authors:** P. Krishnankutty Nair, M. Corredig

**Affiliations:** Food Science Department, University of Guelph, Guelph, ON, Canada N1G 2W1

## Abstract

Concentrated casein micelle suspensions show an altered balance between the colloidal and soluble phases compared with native skim milk. The objective of this research was to probe the role of such a change on the chymosin-driven destabilization of casein micelles. Skim milk was gently concentrated by osmotic stressing to increase the volume fraction of the micelles while maintaining a constant ionic composition. In situ turbidity measurements demonstrated that the secondary stage of gelation was hindered in the concentrated suspensions. Addition of ionic calcium overcame this inhibition, whereas restoring the original concentration by redilution did not. This work clearly demonstrated that calcium plays a major role in decreasing electrostatic repulsion in casein micelles, but also showed the importance of noncolloidal proteins in altering the gelling functionality of casein micelles in concentrated milk. Additional calcium induces aggregation of these soluble caseins, restoring gelation in concentrates.

## Highlights

• In concentrated milk, the secondary stage of renneting is inhibited by addition of soluble caseins.
• Concentrated milk when rediluted with permeate also exhibited impaired renneting functionality.
• This inhibitory effect was attributed to the chelation of calcium by soluble caseins and can be prevented by addition of excess amounts of ionic calcium.

## Graphical Abstract

**Figure:**
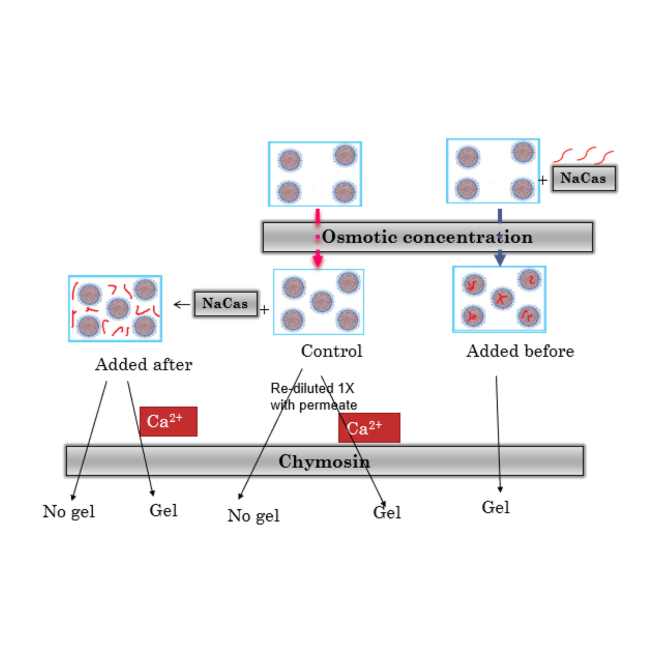
**Summary:** Chymosin-specific activity on κ-casein causes aggregation of casein micelles. The presence of soluble caseins inhibits the secondary stage of this aggregation. This is shown in both rediluted and concentrated samples. The effect can be mitigated by the presence of additional calcium, which can drive soluble caseins to form aggregates. This work yields new evidence of the inhibitory effect of free caseins on rennet-induced gelation of concentrated milk and the role played by added calcium in overcoming such an effect.

The concentration of milk may alter the processing functionality of casein micelles because of the increased protein–protein interactions at high volume fractions (Bouchoux et al., 2009; Krishnankutty Nair et al., 2014), the changes in equilibrium between soluble and colloidal ions (especially calcium), and the increase in soluble (nonsedimentable) calcium (Krishnankutty Nair and Corredig, 2015). The microstructural properties of casein gels are related not only to the internal and interfacial integrity of the casein micelles, but also to the composition of the serum phase, because ionic calcium is critical to the formation of the gel network (Sandra et al., 2012).

Chymosin (rennet)-induced gelation of casein micelles is a functional property of key importance in dairy processing. During this reaction, chymosin specifically hydrolyzes the κ-casein moieties on the surface of casein micelles and cleaves the protective layer of these colloidal particles, inducing their destabilization (Dalgleish, 1992; de Kruif, 1992). In skim milk, when more than 90% of κ-casein is hydrolyzed (Dalgleish, 1992), the micelles aggregate if sufficient calcium ions are present (Lucey, 2009). If additional calcium is added to skim milk, coagulation of casein micelles starts earlier than the 90% threshold (Sandra et al., 2012) because of a reduction in electrostatic repulsion forces.

Studies using milk concentrated by ultrafiltration have demonstrated no difference in the extent of κ-casein hydrolysis needed to reach the point of destabilization (Sandra et al., 2011), despite the increased ratio of casein micelles to free calcium. However, the curd firming rate, which represents the second stage of chymosin-induced aggregation, increases due to the higher frequency of collisions and associations between the micelles (Sandra et al., 2011). Nonetheless, free calcium ions play a role in aggregation—it has been shown with reconstituted milk concentrates that gelation does not occur below a critical concentration of free calcium ions (Martin et al., 2010).

The presence of soluble caseins hinders rennet-induced aggregation of casein micelles in skim milk. The mechanism has been described using highly diluted suspensions in milk serum (Gaygadzhiev et al., 2012), in which calcium ions were not a limiting factor. Similarly, a 3× concentrated milk with added sodium caseinate shows hindered gelation (Krishnankutty Nair and Corredig, 2015). The current study aimed to better understand the role of soluble caseins and calcium in the rennet-induced gelation of concentrated casein micelle suspensions. Concentration was carried out using osmotic stressing, a noninvasive concentration method, maintaining the ionic composition present in the serum of the original milk. We hypothesized that by controlling the level of ionic calcium, it is possible to induce aggregation of the soluble caseins, and thereby restore the aggregation behavior of the casein micelles.

To test this hypothesis, raw milk (obtained from the University of Guelph Dairy Research Station, Guelph, ON, Canada), containing 0.2 g/L sodium azide was centrifuged at 4,000 × *g* for 25 min at 4°C (J2-21 centrifuge, Beckman Coulter) and filtered 4× through a fiberglass filter (Fisher Scientific) to obtain skim milk. Permeate was obtained from ultrafiltration of reconstituted skim milk powder (10% solids, Gay Lea Foods Cooperative) as previously described (Krishnankutty Nair and Corredig, 2015). The permeate contained ~20 m*M* Na^+^, ~40 m*M* K^+^, ~10 m*M* Ca^2+^, ~30 m*M* Cl^−^, ~10 m*M* phosphate, and ~10 m*M* citrate (Lucey and Horne, 2009).

Skim milk was concentrated to 3× using osmotic stressing at 4°C for 18 h (Krishnankutty Nair et al., 2014), using a cellulose dialysis membrane (Spectra/Por 1, 6–8 kDa, Fisher Scientific) and 80 g/L polyethylene glycol (Fluka) in permeate as a stressing agent. The permeate ensured the chemical potential of all ions across the membrane. All experiments were carried out in triplicate (i.e., 3 separate milk batches), and means and standard errors are reported. Statistical significance was evaluated using one-way ANOVA at *P* < 0.05.

Colloidal fractions were separated by centrifuging at 100,000 × *g* for 1 h at 20°C (Beckman Coulter Optima LE-80K). The composition of soluble caseins was then tested using ion exchange chromatography (Krishnankutty Nair and Corredig, 2015). Soluble calcium was measured using nonsuppressed ion chromatography (Rahimi Yazdi et al., 2010) by analyzing the total calcium present in the centrifugal supernatant.

Casein molecules are highly phosphorylated and bind calcium ions via their phosphoserine residues. It has been previously reported that when present in a calcium solution, they form nonsedimentable small particles approximately 12 nm diameter made up of 15 casein molecules (HadjSadok et al., 2008; Pitkowski et al., 2008). The presence of additional soluble casein in the milk may affect the ionic equilibrium between the colloidal and the continuous phase. To determine the distribution of free calcium in different sodium caseinate (**NaCas**) solutions, concentrations ranging from 0 to 10 g/L were dissolved in milk permeate stirred for 2 h. The samples were then filtered using a Prep/Scale-TEF 1ft^2^ cartridge ultrafiltration unit (10 kDa cut-off, regenerated cellulose, Millipore). The soluble caseins were retained in the retentate, and the permeate contained protein-free serum. The permeate was then subjected to calcium analysis to determine the amount of free calcium, which was subtracted from the total calcium present in the solution. Figure 1 indicates the relationship between the amount of soluble caseins added and the residual permeable calcium. We concluded that the addition of 0.6% NaCas to the serum phase would result in further chelation of 3.34 m*M* calcium.

**Figure 1:**
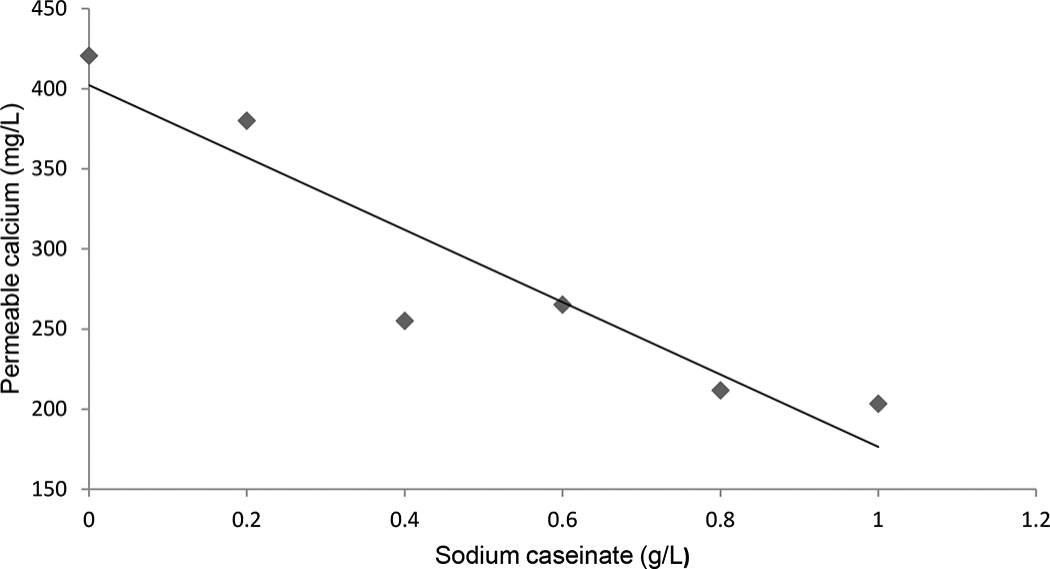
Amount of calcium recovered in the permeable fraction in sodium caseinate solutions dispersed in permeate. Solid line represents the fit; R^2^ = 0.88.

Milk concentrated 3× by osmotic stressing had a serum calcium concentration of 10.23 ± 0.50 m*M*, in agreement with previous literature data for skim milk (Lucey and Horne, 2009). To test the effect of soluble caseins on chymosin-induced gelation, 6 g/L NaCas (Alanate 180, Fonterra New Zealand) was added to the concentrate and stirred for 2 h at room temperature. In addition, CaCl_2_ was added at a final concentration of 3.34 m*M* to the concentrated suspensions containing NaCas, to ensure that sufficient free calcium was present in the system. This amount corresponded to the level chelated by the 6 g/L NaCas added in the concentrate, as shown in Figure 1. These samples showed significantly higher levels of serum calcium, 12.48 ± 0.50 m*M* (*P* < 0.01), compared with the control 3× concentrate with no calcium added.

The gelation behavior of the suspensions was studied using diffusing wave spectroscopy and rheology (Sandra et al., 2012; Krishnankutty Nair and Corredig, 2015). Samples were measured at 30°C after addition of 0.03 international milk clotting units (IMCU)/mL chymosin (Chymax Ultra, Chr. Hansen). Figure 2 shows the changes occurring to light scattering and rheological parameters measured during renneting of concentrated milk and the role of added calcium.

**Figure 2:**
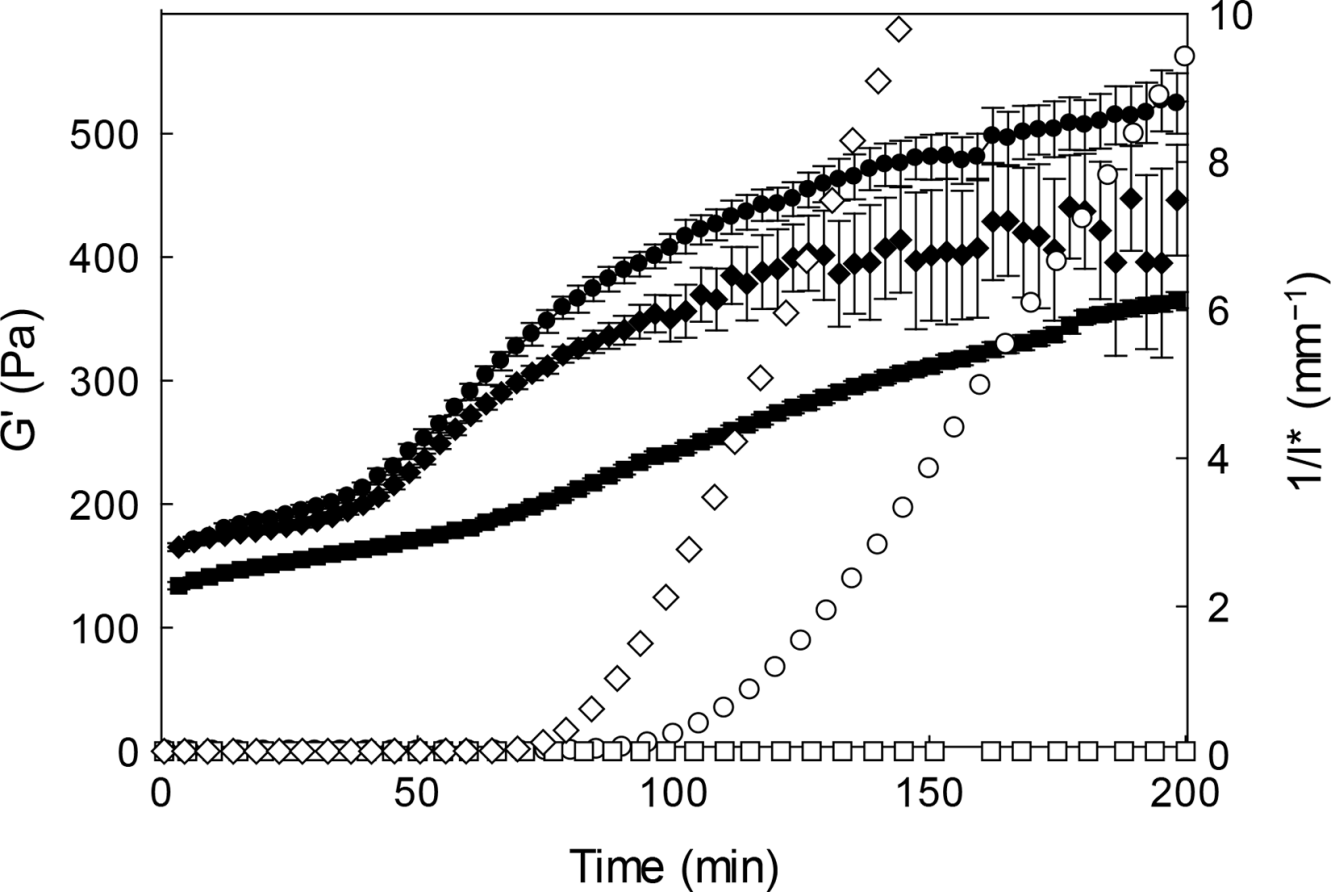
Development of the elastic modulus (empty symbols) and turbidity parameter (1/l*, solid symbols) measured by rheology and diffusive wave spectroscopy for samples after the addition of chymosin. Samples: 3× concentrated milk (circles), same milk with added sodium caseinate after concentration (squares), and the same milk with added sodium caseinate and concentrated with additional calcium chloride (diamonds). The error bars represent the standard error of the turbidity parameter recorded during the experiment.

The development of the elastic modulus (G′, empty symbols in Figure 2) clearly showed that although the 3× concentrated suspension had a gelation point at 100 min, samples with added NaCas did not form stiff gels. The gelation point occurred earlier, at 70 min, when calcium chloride was added to the NaCas suspensions (compared with the control concentrate). We hypothesize that, similarly to what has been noted for skim milk (Sandra et al., 2012), the additional calcium shields the charges and decreases charge repulsion between the micelles. However, a decrease of free ionic calcium by chelation from the additional NaCas does not fully explain the lack of gelation observed in Figure 2.

Turbidity parameters are also shown in Figure 2, as measured by diffusing wave spectroscopy. The 1/l* parameter indicated the start of micellar interactions, which occurred at an earlier stage than the gelation point, as previously shown (Sandra et al., 2012), and, in the case of skim milk, was not affected by the addition of calcium ions. In the 3× concentrated control, an inflection point in 1/l* can be observed at about 42 min after the addition of chymosin. The inflection point occurred later for samples containing NaCas, demonstrating that the presence of the NaCas hindered particle–particle interactions. When calcium chloride was added to the NaCas suspensions, the 1/l* inflection point was fully restored and occurred at the same time as in the original concentrate. We concluded that addition of calcium caused aggregation of the soluble caseins, which in turn impaired their stabilizing behavior.

To further confirm the role of soluble caseins in aggregation, we restored the concentrated suspensions to the original casein-to-serum calcium ratio by redilution with milk serum (permeate). Regardless of the treatment, none of the samples showed development of a gel modulus (data not shown). All samples showed the presence of dissociated caseins, as clearly demonstrated by analysis of the centrifugal supernatants (Figure 3).

**Figure 3:**
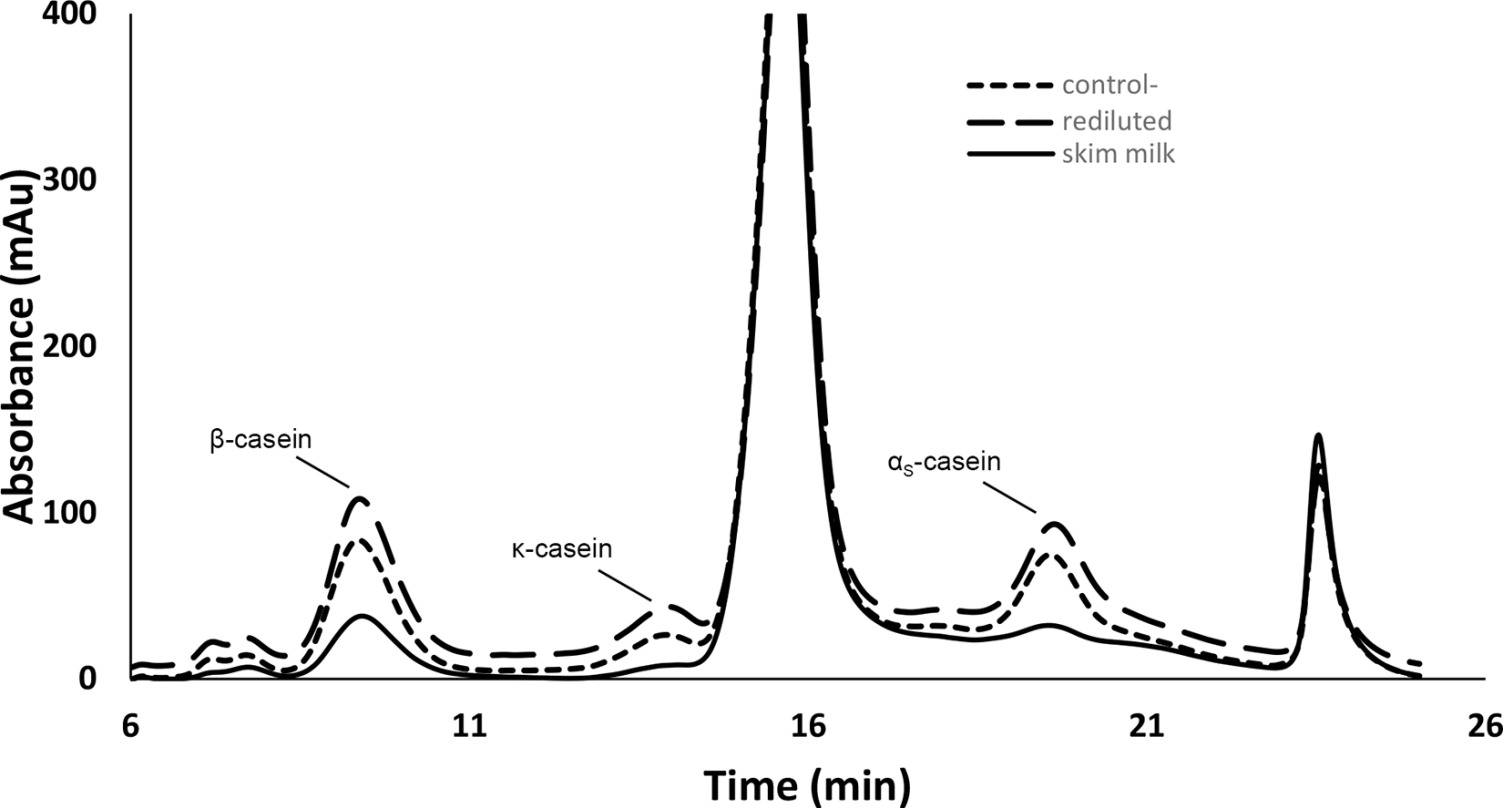
Ion exchange chromatography of the centrifugal supernatants of skim milk (control; solid line), 3× concentrate rediluted with permeate (dotted line), and 3× concentrate with added sodium caseinate, rediluted in permeate (dashed line). Whey proteins eluting at 15 min (large peak) and main casein fractions are indicated in the graph.

Figure 3 shows the composition of the centrifugal supernatants of skim milk and of the 3× concentrate with or without additional sodium caseinate after redilution with permeate. All of the rediluted samples showed higher peaks for caseins than the serum phase of the original milk, confirming that the casein micelles contained much higher level of soluble caseins upon redilution. The centrifugal supernatants contained higher amounts of β-casein, but also of α_S_-caseins, implying the dissociation of micelles with redilution. All samples showed similar levels of whey proteins (eluting at 15 min).

The altered renneting functionality of casein micelles after concentration can be attributed to the presence of an increased amount of soluble caseins. This may in part affect the amount of ionic calcium present in the serum; however, we can also conclude that the limited aggregation cannot be attributed to calcium chelation. There is a threshold limit for the amount of monomeric proteins the micelles can hold without affecting the rennet functionality of casein micelles. In the presence of calcium, caseins may form small colloidal particles. At a sufficiently high concentration of calcium, these larger colloidal structures may co-precipitate with casein aggregates during renneting. When an excess amount of monomeric proteins is present, aggregation is hindered by associating it with the casein micelles and providing additional steric repulsion to rennet-altered casein micelles.
